# Supplementary material for: A quantized mechanism for activation of pannexin channels
Source: Nat Commun. 2017 Jan 30;8:14324. doi: 10.1038/ncomms14324 (PMC5290276; doi:10.1038/ncomms14324)
Supplement: Supplementary Information — Supplementary figures and Supplementary table. [file ncomms14324-s1.pdf]

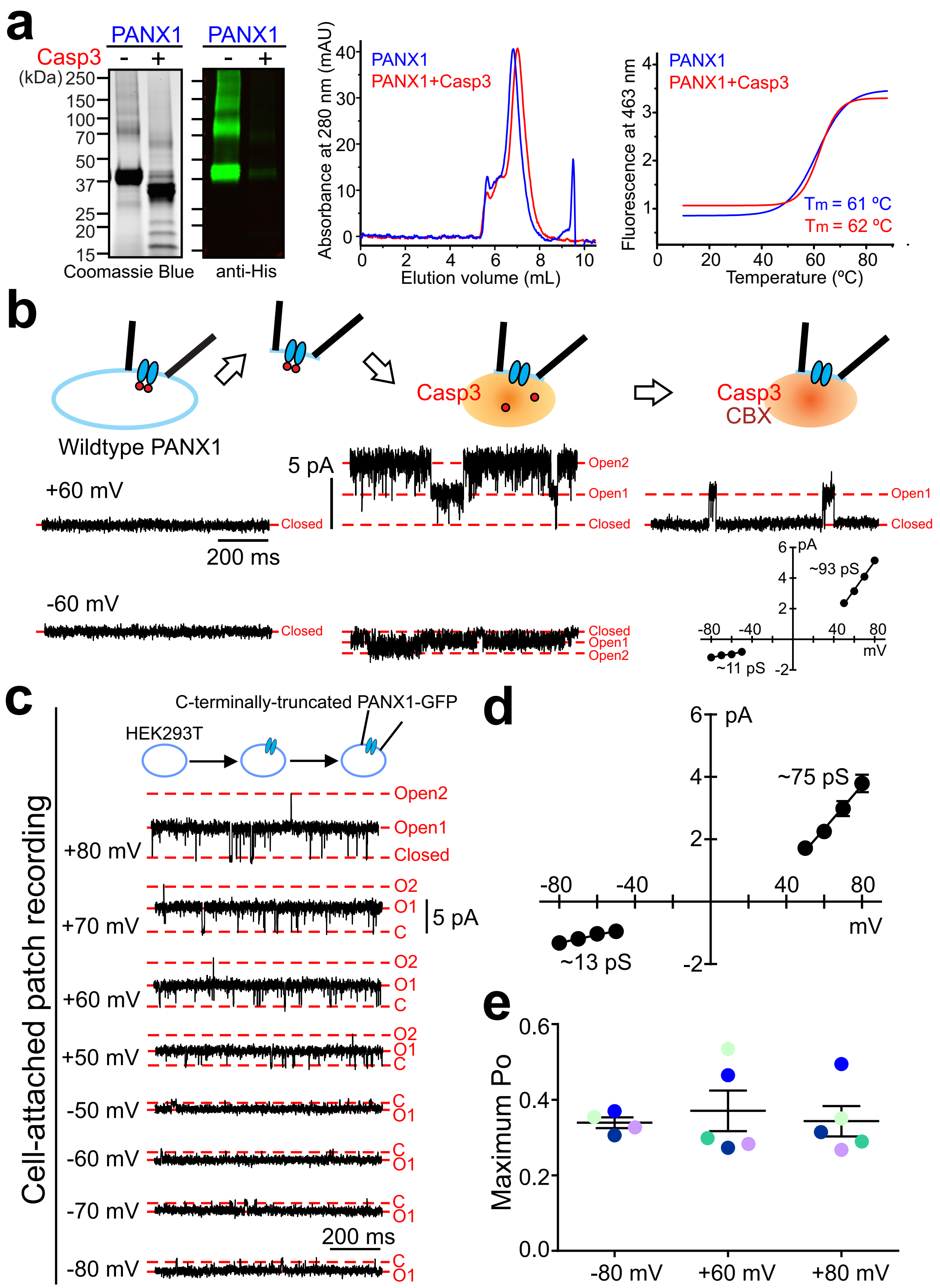
a
PANX1
Casp3
- +
(kDa) 250
150
100
70
50
37
25
20
15
Coomassie Blue
anti-His
Absorbance at 280 nm (mAU)
PANX1
PANX1+Casp3
Elution volume (mL)
Fluorescence at 463 nm
Tm = 61 ºC
Tm = 62 ºC
Temperature (ºC)
b
Wildtype PANX1
Casp3
CBX
5 pA
+60 mV
-60 mV
200 ms
Open2
Open1
Closed
~93 pS
~11 pS
pA
mV
c
C-terminally-truncated PANX1-GFP
HEK293T
Cell-attached patch recording
+80 mV
+70 mV
+60 mV
+50 mV
-50 mV
-60 mV
-70 mV
-80 mV
O2
O1
C
5 pA
200 ms
d
~75 pS
~13 pS
pA
mV
e
Maximum Po
-80 mV
+60 mV
+80 mV

Supplementary Figure 1. Properties of purified PANX1 and single-channel properties of C-terminally truncated PANX1

(**a**) Purified PANX1 protein was cleaved *in vitro* by caspase 3 (Casp3). Coomassie Blue stained gel showed full-length PANX1 (~48 kDa), and caspase-cleaved PANX1 (~42 kDa). Immunoblot probed with anti-histidine antibody showed diminished signal for Casp3-cleaved PANX1 due to removal of the C-terminal histidine tag. Size-exclusion chromatographs (middle) and fluorescent thermal stability assay (right) showed both purified full-length PANX1 (blue) and Casp3-cleaved PANX1 (red) are homogenous and thermally stable. (**b**) Inside-out patch recording performed in HEK293T cells expressing wildtype, untagged PANX1. Examples of PANX1 single-channel activity recorded at +60 mV (upper) or -60 mV (lower), before (left) and after (middle) caspase 3 exposure, and after carbenoxolone (CBX, 50 μM) inhibition. Current-voltage (I-V) relationship (lower right) demonstrates that Casp3-cleaved wildtype PANX1 has unitary conductance of 93.0 ± 4.8 pS (at +50~+80 mV, n=3) and 11.4 ± 1.2 pS (at -80~-50 mV, n=3) (**c**) Examples of PANX1 single-channel activity obtained by cell-attached recording from HEK293T cells expressing C-terminally-truncated PANX1, with a GFP tag. C, closed state. O, open state. (**d**) I-V relationship obtained from cell-attached recordings demonstrates that C-terminally-truncated PANX1 has unitary conductance of 74.7 ± 3.6 pS (at +50~+80 mV, n=8) and 12.8 ± 1.2 pS (at -80~-50 mV, n=3). (**e**) Maximum Po of C-terminally-truncated PANX1 is similar across different patch potentials (-80, +60, and +80 mV). Data obtained from the same patch is labeled in the same color.

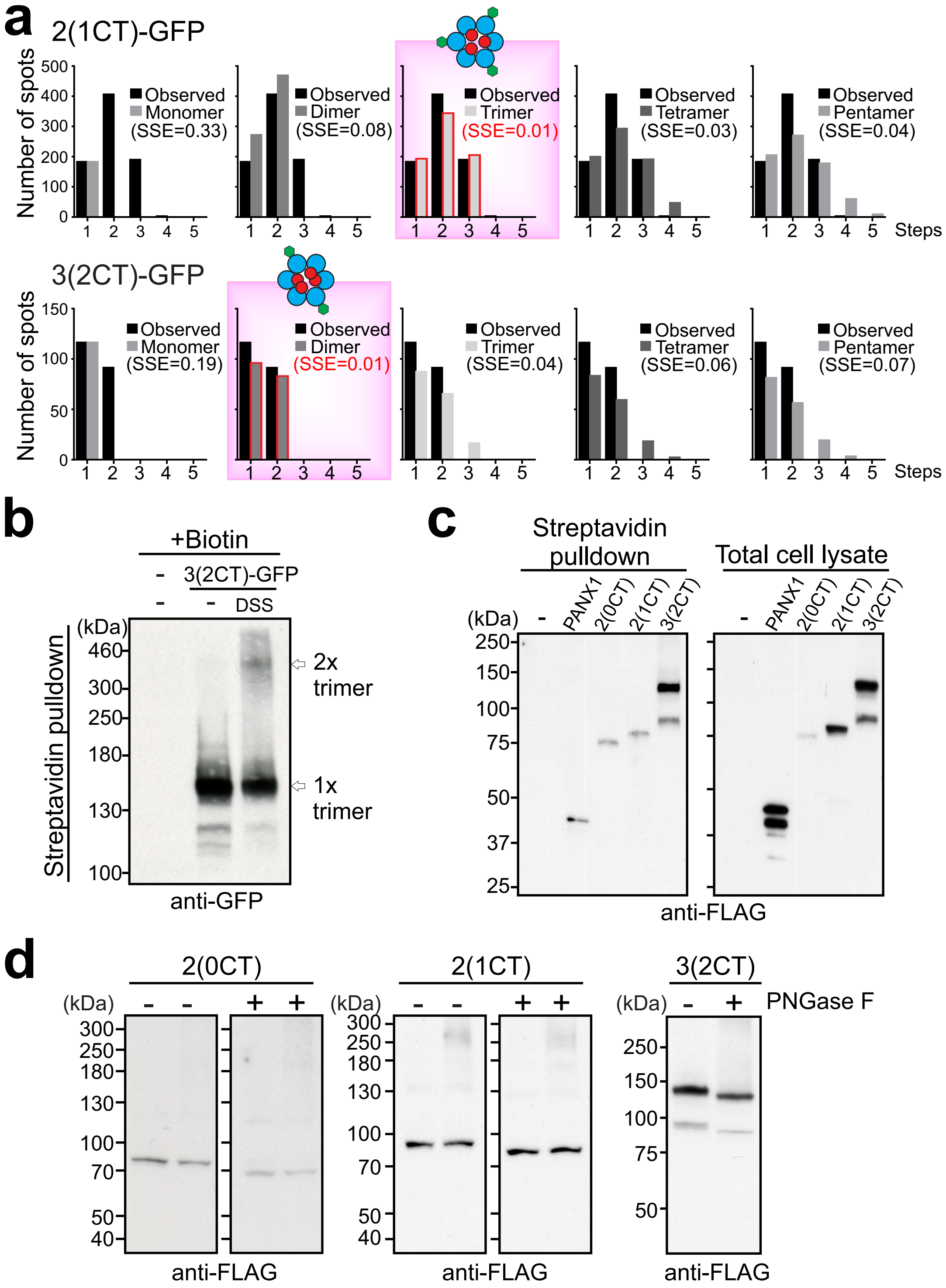
a
2(1CT)-GFP
Number of spots
500
400
300
200
100
0
Observed
Monomer
(SSE=0.33)
Observed
Dimer
(SSE=0.08)
Observed
Trimer
(SSE=0.01)
Observed
Tetramer
(SSE=0.03)
Observed
Pentamer
(SSE=0.04)
1 2 3 4 5
Steps
3(2CT)-GFP
Number of spots
150
100
50
0
Observed
Monomer
(SSE=0.19)
Observed
Dimer
(SSE=0.01)
Observed
Trimer
(SSE=0.04)
Observed
Tetramer
(SSE=0.06)
Observed
Pentamer
(SSE=0.07)
1 2 3 4 5
Steps
b
+Biotin
-
3(2CT)-GFP
-
-
DSS
(kDa)
460
300
250
180
130
100
Streptavidin pulldown
2x trimer
1x trimer
anti-GFP
c
Streptavidin pulldown
Total cell lysate
(kDa)
-
PANX1
2(0CT)
2(1CT)
3(2CT)
250
150
100
75
50
37
25
anti-FLAG
d
2(0CT)
2(1CT)
3(2CT)
(kDa)
- - + +
PNGase F
300
250
180
130
100
70
50
40
150
75
anti-FLAG
anti-FLAG
anti-FLAG

Supplementary Figure 2. PANX1 concatemers suggest a hexameric channel stoichiometry at the cell surface

(**a**) Distribution of observed photobleaching events from HEK293T cells expressing 2(1CT)-GFP (upper panels, black bars) or 3(2CT)-GFP (lower panels, black bars). Best fit binomial distributions (SSE provided) for each indicated stoichiometry (gray bars) with percentage of fluorescent GFP allowed to vary. Panels shaded in red represent the lowest SSE among different distributions. (**b**) Dimeric (2x) and monomeric (1x) forms of 3(2CT)-GFP were found in cross-linked and cell-surface biotinylated samples, supporting hexameric channel conformation of PANX1 at the cell surface. HEK293T cells expressing 3(2CT)-GFP were first labeled with membrane impermeable biotin, followed by exposure to the membrane permeable crosslinker, DSS. Biotinylated proteins were precipitated using streptavidin-agarose beads and detected with anti-GFP antibody. (**c**) Representative cell-surface biotinylation assay (from n=3) showed that the full-length monomeric and the concatenated PANX1 channels, 2(0CT), 2(1CT), and 3(2CT), were expressed at the cell surface, primarily at the predicted molecular weights (~80, ~90, or ~140 kDa). PANX1 concatemers were detected with anti-FLAG antibody. Left: FLAG-tagged PANX1 concatemers from streptavidin-pulldown samples. Right: FLAG-tagged PANX1 concatemers from total cell lysates. Note that although proteins with size of ~100 kDa were detected in 3(2CT)-expressing cells, the larger ~140-kDa protein was predominantly expressed at the cell surface. (**d**) Dimeric and trimeric PANX1 concatemers are glycosylated. HEK293T cells were transiently transfected with 2(0CT), 2(2CT), or 3(2CT), and whole cell lysates were treated with 50% glycerol or PNGase F (7000 $U \cdot mg^{-1}$), followed by SDS-PAGE analysis using anti-FLAG antibodies to detect concatemers.

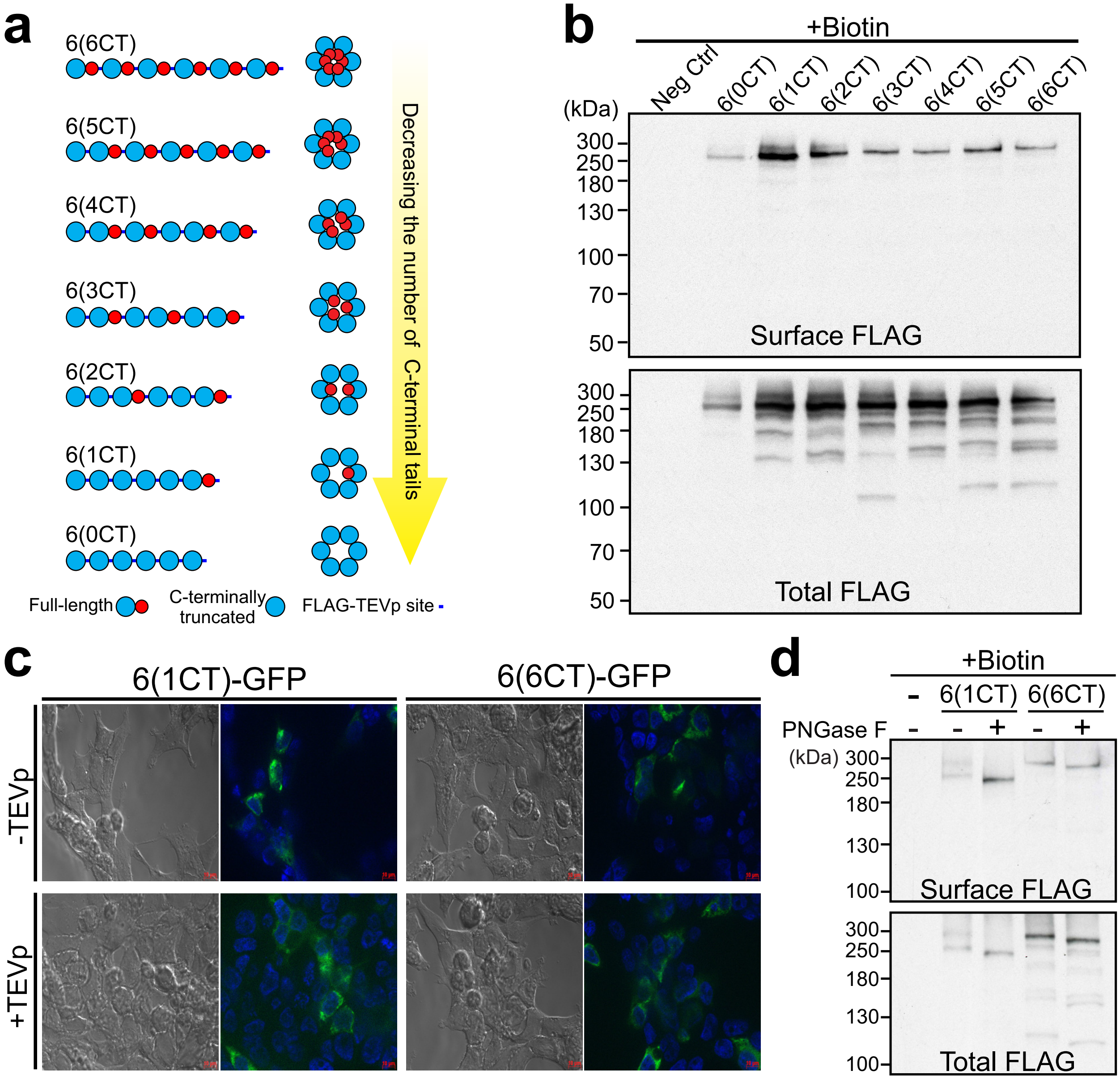

Supplementary Figure 3. Hexameric PANX1 concatemers are glycosylated and expressed at the cell surface

(**a**) Schematic shows a panel of hexameric PANX1 concatemers containing varying number of full-length or C-terminally-truncated subunits. (**b**) Representative cell-surface biotinylation (from n=3) showed all hexameric PANX1 concatemers are expressed at the cell surface at their predicted molecular weights (from ~240-300 kDa). PANX1 concatemers were detected with anti-FLAG antibody. Upper: FLAG-tagged PANX1 concatemers from streptavidin-pulldown samples. Lower: FLAG-tagged PANX1 concatemers from whole cell lysates. Note that the lower molecular weight proteins found in total cell lysates were not detected in streptavidin-pulldown samples. (**c**) Expression of 6(1CT)-GFP and 6(6CT)-GFP in HEK293T cells, with or without TEVp co-expression. Bright field (left) and fluorescent (right) images were taken using 63x Axio Imager 2. Green: GFP-tagged PANX1 concatemers. Blue: cell nuclei stained by Hoechst 33342. Scale bar: 10 μm. (**d**) Hexameric PANX1 concatemers are glycosylated. HEK293T cells were transiently transfected with 6(1CT) or 6(6CT), followed by exposure to membrane-impermeable biotin; cells were then lysed, and cell lysates were treated with 50% glycerol or PNGase F (7000 U·mg^{-1}), followed by streptavidin pull-down. Upper: FLAG-tagged PANX1 concatemers from streptavidin pull-down samples. Lower: FLAG-tagged PANX1 concatemers from whole cell lysates.

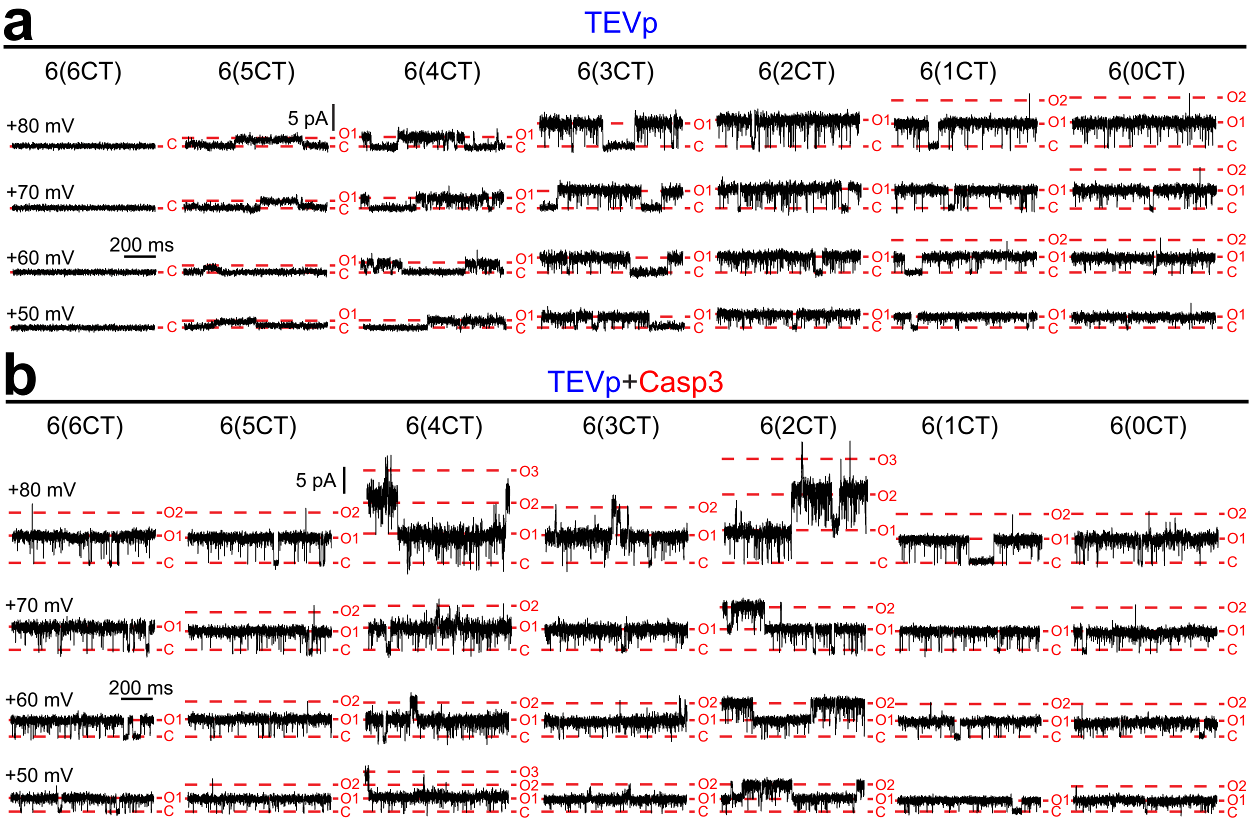

Supplementary Figure 4. Single-channel activity of hexameric PANX1 concatemers activated by exposure to TEVp and Casp3 in inside-out patch recordings

Exemplar inside-out patch recordings obtained at +50, +60, +70, and +80 mV from HEK293T cells expressing hexameric PANX1 concatemers. (**a**) Steady-state single-channel activities after TEVp exposure. (**b**) Steady-state single-channel activities from the same inside-out patches after Casp3 exposure.

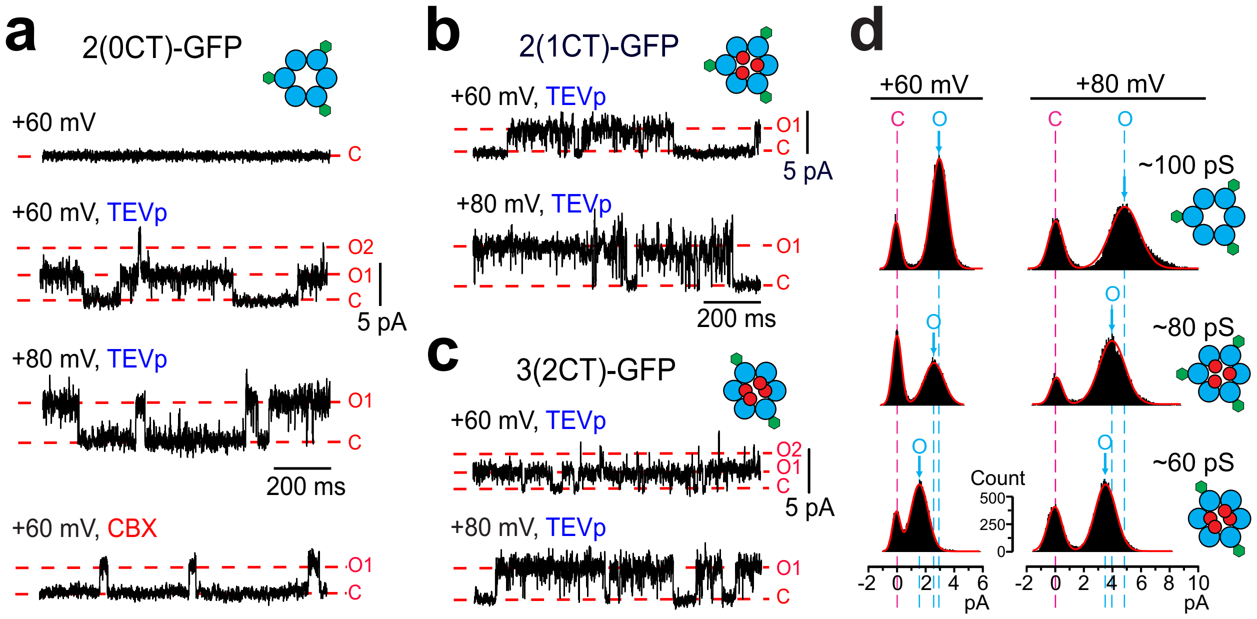

Supplementary Figure 5. Inside-out patch recordings of functional GFP-tagged dimeric and trimeric PANX1 concatemers show increasing conductance with fewer intact C-termini

(**a**-**c**) Inside-out patches excised from HEK293T cells expressing concatenated PANX1 constructs were silent prior to TEVp exposure (**a**, top). After TEVp-mediated cleavage of the inter-subunit linkers, single-channel activity was observed from 2(0CT)-GFP (**a**), 2(1CT)-GFP (**b**), and 3(2CT)-GFP (**c**) channels at +60 or +80 mV. Single-channel activity was reduced by carbenoxolone (CBX, 50 μM) (**a**, bottom). (**d**) Histograms show that unitary currents, obtained at +60 and +80 mV after TEVp treatment, increased in graded fashion as the numbers of C-terminal tails were reduced. Data are representative from n=2 for each constructs.

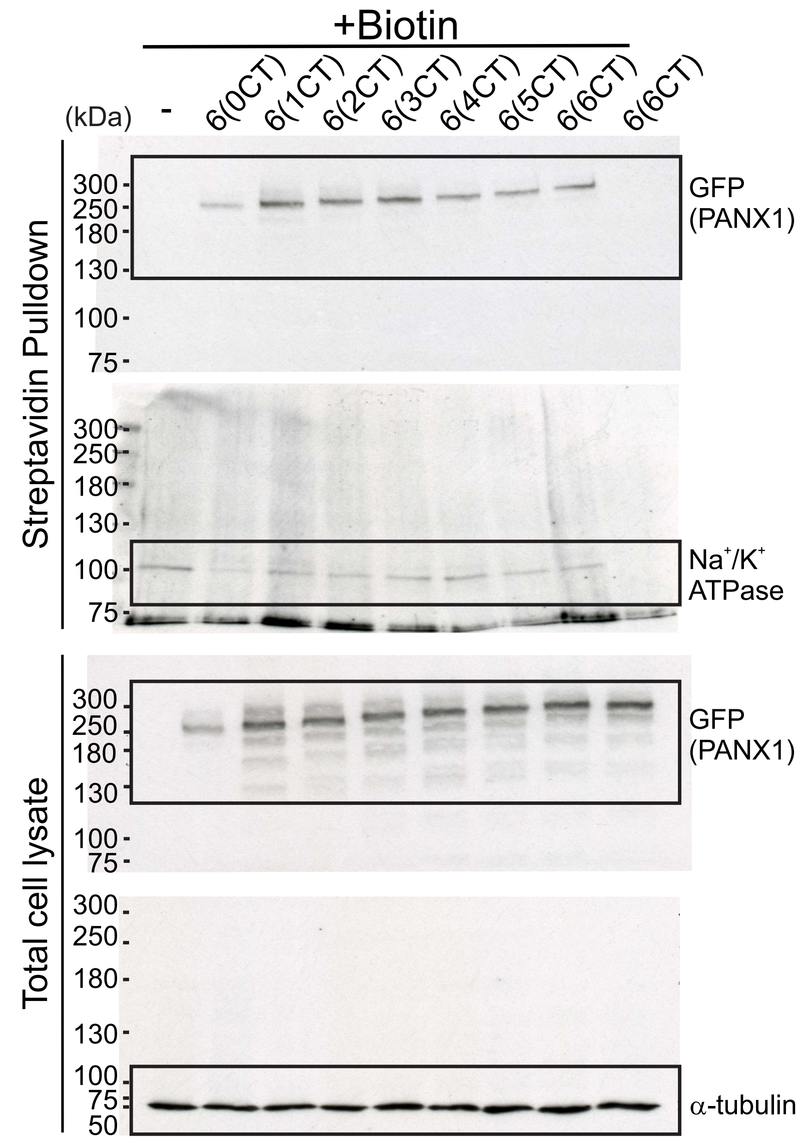

Supplementary Figure 6. Uncropped images of Western blots presented in Figure 5b.
The boxed regions are those shown in Figure 5b of the paper.

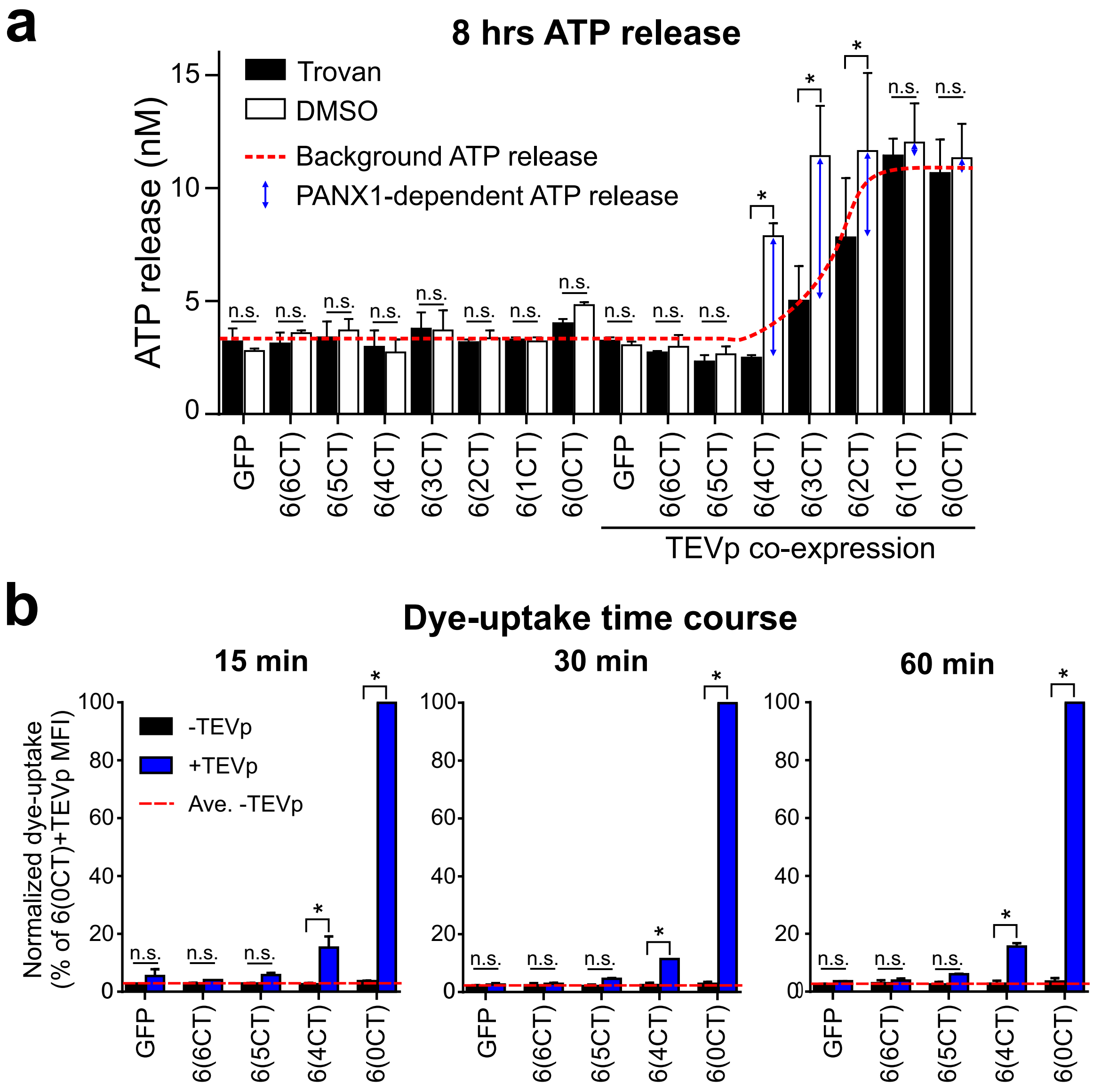

Supplementary Figure 7. Removing at least two PANX1 C-tails is required for ATP or dye permeation.

(**a**) ATP release from HEK293T cells expressing GFP-tagged hexameric PANX1 concatemers, with or without TEVp co-expression. Cells were incubated in assay buffer containing ARL67156 (300 µM) for 8 h, with either DMSO (white) or trovafloxacin (Trovan, 25 µM, black). Vertical double arrows (blue) indicate PANX1-dependent (i.e., Trovan-sensitive) ATP release. Red dashed line represents trend of background ATP release (i.e., Trovan-*in*sensitive and PANX1-*in*dependent). Note that increasing background ATP accumulation was observed with more active hexameric constructs under these conditions, likely reflecting cell death, and making this longer time point unreliable for quantitative comparisons across all constructs. (**b**) TO-PRO-3 uptake for 15, 30, or 60 min in Jurkat cells expressing GFP-tagged hexameric PANX1 concatemers. Cells were co-expressed with (blue) or without (black) TEVp. Red dash lines depict the averaged TO-PRO-3 uptake from cells without TEVp co-expression. Data are presented as mean ± s.e.m. from two independent experiments. *: P<0.05 using two-way ANOVA followed by Fisher's LSD test; n.s.: no statistical significance.

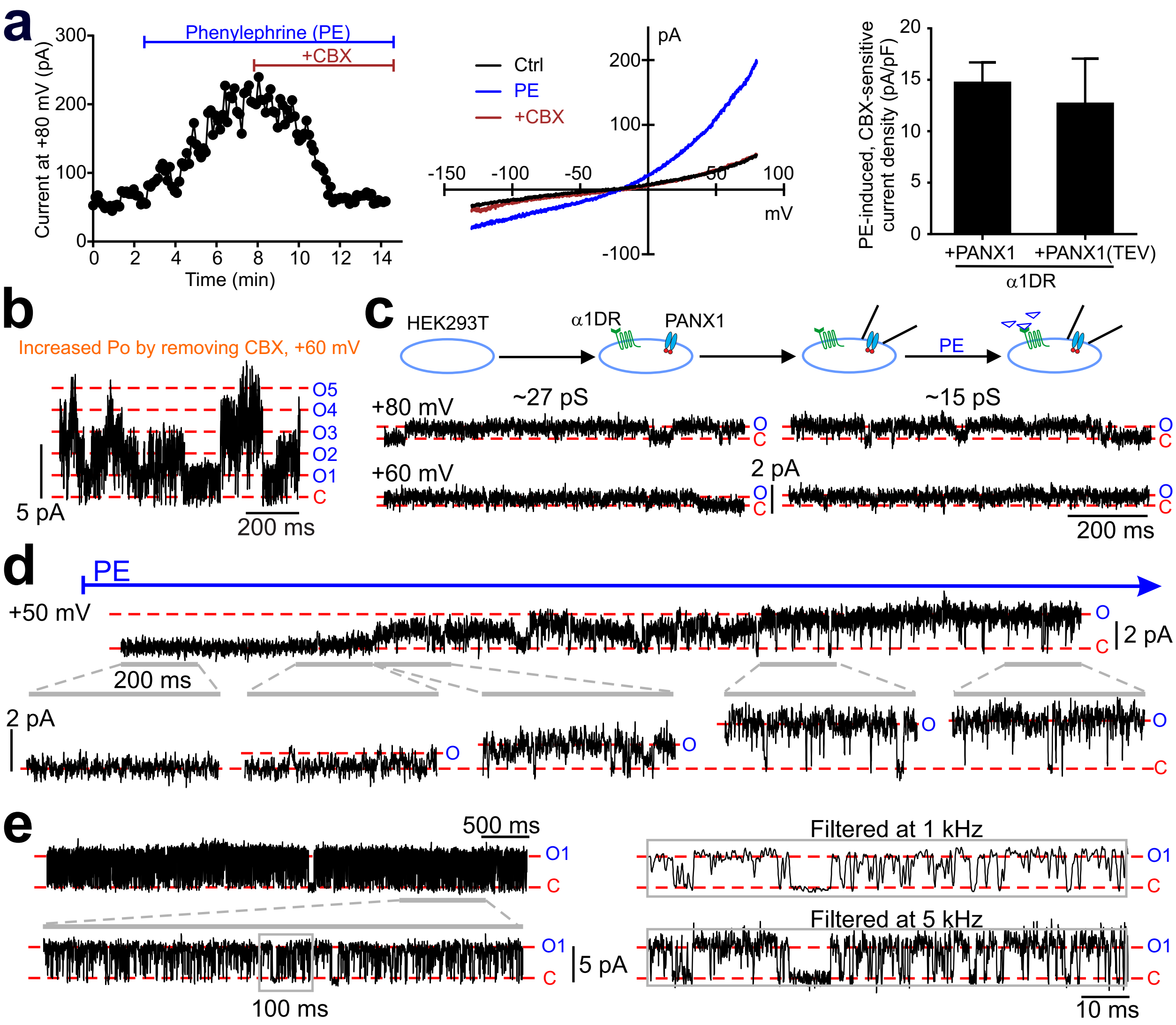

Supplementary Figure 8. α1DR-mediated activation of PANX1 channels

(**a**) Whole-cell recordings obtained from HEK293T cells heterologously expressing α1D adrenoceptor (α1DR) along with FLAG-tagged full-length PANX1 or PANX1(TEV). Left: whole-cell PANX1 current (at +80 mV) was increased by phenylephrine (PE, 20 μM) and inhibited by CBX (50 μM). Middle: I-V relationships under control conditions (Ctrl, black), during phenylephrine stimulation (PE, blue) and after CBX inhibition (brown). Right: averaged current density induced by PE is 14.7 ± 2.0 pA/pF for full-length PANX1 (n=6), and 12.6 ± 4.4 pA/pF for PANX1(TEV) (n=3). Note that PANX1(TEV) is caspase-cleavage resistant. (**b**) Increased activity of five α1DR-activated PANX1 channels after removing CBX inhibition (from the same patch shown in Fig. 6c). (**c**) Examples of PE-activated PANX1 channels with small conductance (~27 pS for left and ~15 pS for right) obtained from different patches at +80 and +60 mV by cell-attached recording. (**d**) Examples of cell-attached recordings during PE stimulation from a HEK293T cell expressing α1DR and GFP-tagged full-length PANX1. Single-channel activity shows gradually increased single-channel current amplitude at +50 mV over the initial period of PE stimulation (from left to right). (**e**) Cell-attached recording of steady-state PANX1 activation induced by PE stimulation. Exploded views from the gray-boxed 100-ms are filtered at 1 kHz (upper-right) or 5 kHz (lower-right).

Supplementary Table 1. Primers used for PANX1 cloning

Primer	Sequence (5’ to 3’)	Note
#1	5'-GATGGCCATCGCTCAACTGGC-3’	Forward primer for subcloning full-length, wildtype PANX1 into pVL1392
#2	5'-CTAGTGGTGGTGGTGGTGGTGCTTGTCGTCGTCATCGCAAGAAGAATCCAGAAGTCTCT-3’	Reverse primer for subcloning full-length, wildtype PANX1 into pVL1392
#3	5'-TCACTAGTCGCGGCCATGGCCATCGCTCAACTGGCC-3’	Forward primer for subcloning full-length His-tagged PANX1 into pFastBac1
#4	5’-ACTTCTCGACAAGCTCTAGTGGTGGTGGTGGTGGTG-3’	Reverse primer for subcloning full-length His-tagged PANX1 into pFastBac1
#5	5'-GACTTCTGGATTCTTCTTGCTAATGAGGTACCGACGGCCGCGGAG-3'	Forward primer for removing FLAG tag from full-length PANX1-FLAG
#6	5'-CTCCGCGGCCGTCGGTACCTCATTAGCAAGAAGAATCCAGAAGTC-3'	Reverse primer for removing FLAG tag from full-length PANX1-FLAG
#7	5'-CTGGATCGACTAGTGTCGACATGGCCATCGCTC-3'	Forward primer for inserting SalI site at 5' of full-length or C-terminally truncated PANX1-FLAG
#8	5'-GAGCGATGGCCATGTCGACACTAGTCGATCCAG-3'	Reverse primer for inserting SalI site at 5' of full-length or C-terminally truncated PANX1-FLAG
#9	5'-GTGACTACAAGGACGACGATGACAAGGAGAATTTGTATTTCCAAGGTCTCGAGTGACGGCCGCGACTCTAGAGTGAGGG-3'	Forward primer for inserting TEVp recognition site and XhoI site at 3' of full-length PANX1-FLAG
#10	5'-CCCTCACTCTAGAGTCGCGGCCGTCACTCGAGACCTTGGAAATACAAATTCTCCTTGTCATCGTCGTCCTTGTAGTCAC-3'	Reserve primer for inserting TEVp recognition site and XhoI site at 3' of full-length PANX1-FLAG
#11	5'-GGCGACTACAAGGACGACGATGACAAGGAGAATTTGTATTTCCAAGGTCTCGAGTGAGGTACCGACGGCCGCGGAGCTGGTG-3'	Forward primer for inserting TEVp recognition site and XhoI site at 3' of C-terminally truncated PANX1-FLAG
#12	5'-CACCAGCTCCGCGGCCGTCGGTACCTCACTCGAGACCTTGGAAATACAAATTCTCCTTGTCATCGTCGTCCTTGTAGTCGCC-3'	Reverse primer for inserting TEVp recognition site and XhoI site at 3' of C-terminally truncated PANX1-FLAG
#13	5'-GGACGACGATGACAAGGAGACTGTGCGTTTCCAAAGTATGGTGAGCAAGGGCGAGGAGCTGTTCAC-3'	Forward primer for eGFP overlap-extension PCR round 1
#14	5'-CTTCTAGATCACTCGAGCTTGTACAGCTCGTCCATGCCGAGAGTGATC-3'	Reverse primer for eGFP overlap-extension PCR round 1. Reverse primer for PANX1-eGFP overlap-extension PCR round 2
#15	5'-GAGAATTCTGTCGACATGGCCATCGCTCAACTGGCCAC-3'	Forward primer for PANX1 overlap-extension PCR round 1. Forward primer for PANX1-eGFP overlap-extension PCR round 2
#16	5'-GTGAACAGCTCCTCGCCCTTGCTCACCATACTTTGGAAACGCACAGTCTCCTTGTCATCGTCGTCC-3'	Reverse primer for PANX1 overlap-extension PCR round 1
